# Clinical Midwifery

**Published:** 1853-07

**Authors:** Chas. W. Turner

**Affiliations:** Surgeon to the Dispensary, etc., Minchinhampton


*Clinical Midwifery.—Turning; Transfusion.* By Chas. W. Turner, Esq., M. R. C. S., Surgeon to the Dispensary, etc., Minchinhampton.—On Monday, Dec. 13th, I was summoned to see Elizabeth C——, aged thirty-nine, in labor with her ninth child. She was a short, strong-built, muscular woman, and had been in labor many hours. About two months previously two of her children were run over by a wagon, and one of them sustained a fracture of the thigh. She says she thought she should have been confined at that time, as the fright brought on severe labor-pains, attended with a considerable discharge of water. She thinks the shock to her system, in consequence of the accident, caused the situation, or rather the position, of the fœtus to be changed; be that, however, as it may, certain it is, that on my making an examination I discovered the hand presenting. This I returned, and, after some difficulty, I succeeded in bringing down one of the feet, and in due time delivered her of a very large child. As the placenta was not readily thrown off, and as the womb was well contracted, I thought under the circumstances, it would be better to use no force; I therefore left her under the care of the midwife who was in attendance, and desired her, in the event of any symptom occurring requiring my attendance, to dispatch a messenger to me. Hearing nothing of the case after a lapse of several hours, I considered all was going on well, and I proceeded to visit a patient eleven miles off, who was very desirous of seeing me. On my return home in the afternoon, I found several urgent messages had been fotwarded to my house, and in consequence I lost no time in visiting the woman. I was much alarmed at the condition in which I found her. Her countenance was anxious and pallid, her lips bloodless, and not a vestige of pulse to be felt at either wrist; the voice was low, she complained of a load upon her chest, and there was a constant disposition to syncope. In consequence of my absence from home, Mr. Wells kindly supplied my place, and removed the placenta, which was firmly adherent. She had lost a good deal of blood, but this had ceased on my arrival. Mr. Wells had given her brandy, and applied hot bottles to her feet; he said, however, that he could detect no pulse at the time of his visit, neither could I an hour or two later, notwithstanding she had had a good deal of brandy-and-water. It was clear we had to contend with a very desperate case—that life was fast ebbing; and there seemed to be nothing left to be done but the operation of transfusion. Doubtful as the remedy was, Mr. Wells agreed with me in the propriety of trying it. I therefore had up the husband, and explained to him that his wife was sinking from loss of blood, and that the only chance remaing for her was supplying this loss by throwing some blood into her system, and that for this purpose it was requisite that I should abstract some blood from him. To this he consented. Having made an opening into the vein of the right arm of the woman, I took a brass syringe capable of holding about three ounces. This I warmed by means of hot water; I then opened a vein in the arm of the man and received the blood into the syringe. I inserted the ivory point of the syringe; but, perhaps from the opening in the vein being too small, the stream got under the cellular tissue. I therefore cut down upon a vein in the other arm, and, with a syringe full of fresh bood, I directed the stream upwards. This appeared to do very well, and in about a quarter of an hour afterwards Mr. Wells thought he could discover a very faint pulsation. Still her condition was anything but promising; and, indeed, at one time during the operation, both Mr. Wells and myself thought she was gone. When her husband came to her, she said she never had such feelings before, and she was certain she was dying. However, I persevered in giving her hot egg-tea, with brandy, and occasionally port wine, (as she liked the latter the best of the two,) and incessantly applied the smelling bottle to her nostrils, also taking care to keep the feet warm by means of hot water. I also gave her a cordial mixture, containing chloric ether, and which certainly was very useful. She got through the night better than could have been expected, and the next morning she was decidedly better; she spoke more audibly, and there was some strength in the beat of the carotids; the pulse admitted of numbering, but was very weak indeed; and, although much exhausted, that disposition to syncope had entirely disappeared, and the tympanitic state of the bowles, which was so observable yesterday, to-day was much diminished. She passed no urine for thirty-four hours after her delivery, which made me anxious; this secretion, however, was restored, and for four days nothing could be more satisfactory than her progress. On the fifth day, her appetite was good, she felt stronger, and in all respects appeared to be going on well, with the exception of a hard boil on the back of the left forearm. This had been coming for a fortnight previous to her confinement, but had only become troublesome to-day. Each day this became harder and more extensive and inflamed, till at length the limb became so large and painful, from the tip of the fingers to the shoulder-joint, that she could obtain no rest; she became feverish, lost her appetite, her breath got short, and on the 23d of December, ten days from her confinement, she died I should state that I cut deep and completely through the carbuncle, and with decided relief; the pain, after this incision, was much subdued, or, to use her own expression, I had "cut away the pain." There was scarcely any blood followed, and the sensation was that of cutting through brawn.

I should have been glad had the woman lived; nevertheless, she survived sufficiently long for the operation to be pronounced thoroughly successful; her death arose quite from extraneous causes.—*ibid.*

